# Ring-Shaped Piezoelectric 5-DOF Robot for Angular-Planar Motion

**DOI:** 10.3390/mi13101763

**Published:** 2022-10-18

**Authors:** Andrius Čeponis, Dalius Mažeika, Vytautas Jūrėnas, Dovilė Deltuvienė, Regimantas Bareikis

**Affiliations:** 1 Department of Electrical and Electronics Engineering, Technical Faculty, Vilnius College of Technologies and Design, Olandų Str. 16, LT-10223 Vilnius, Lithuania; 2 State Key Laboratory of Mechanics and Control of Mechanical Structures, College of Aerospace Engineering, Nanjing University of Aeronautics and Astronautics, Nanjing 210016, China; 3 Department of Information Systems, Faculty of Fundamental Sciences, Vilnius Gediminas Technical University, Sauletėkio Avn., 11, LT-10223 Vilnius, Lithuania; 4 Robotics and Piezomechanics Laboratory, Institute of Mechatronics, Kaunas University of Technology, K. Donelaičio Str. 73, LT-44249 Kaunas, Lithuania; 5 Department of Mathematical Statistics, Faculty of Fundamental Sciences, Vilnius Gediminas Technical University, Sauletėkio Avn., 11, LT-10223 Vilnius, Lithuania; 6 Department of Mechanical and Material Engineering, Faculty of Mechanics, Vilnius Gediminas Technical University, Sauletėkio Avn., 11, LT-10223 Vilnius, Lithuania

**Keywords:** piezoelectric robot, angular-planar motion, 5-DOF motion

## Abstract

This paper provides numerical and experimental investigations of a ring-shaped piezoelectric 5-DOF robot that performs planar and angular motions of spherical payload. The robot consists of a piezoelectric ring glued on a special stainless-steel ring with three spikes oriented in the radial direction of the ring. The spherical payload is placed on top of the piezoelectric ring and is moved or rotated when a particular excitation regime is used. An alumina oxide ball is glued at the end of each spike of the steel ring and is used as contacting element. The spikes are used to transfer vibrations of the piezoelectric ring to contacting elements and to induce the planar motion of the payload. Additionally, three alumina oxide balls are glued on the top surface of the piezoelectric ring and are used to generate rotational motion of the spherical payload by impacting it. Finally, the top electrode of the piezoceramic ring is divided into six equal sections and is used to control the direction of angular and planar motion of the payload. Numerical modeling of the robot showed that vibration modes suitable for angular and planar motions are obtained at a frequency of 28.25 kHz and 41.86 kHz, respectively. Experimental investigation showed that the maximum angular velocity of the payload is 30.12 RPM while the maximum linear motion of the robot is 29.34 mm/s when an excitation voltage of 200 V_p-p_ was applied and a payload of 25.1 g was used.

## 1. Introduction

Mechatronic systems are used in modern engineering applications such as robots, medical devices, high precision positioning systems as well as other fields where the demand for motion systems with high accuracy is relevant. Accuracy is one of the main features of mechatronic devices, however scalability and size become significant features because they allow for the reduction of dimensions or the integration of them into a particular system. The device must consist of a minimal number of components to reduce the size of the system. Such a type of system allows for increased reliability, controllability, and accuracy of the system. In addition, there is a demand for motion actuation systems that can provide multi-degrees-of-freedom (MDOF) devices [1]. Usually, electromagnetic actuators and motors are used for that purpose. However, such a system includes several single-degree of freedom actuators used to achieve MDOF motion. The application of electromagnetic actuators and motors for multi-degree-of-freedom systems with high accuracy has several drawbacks, such as magnetization of payload or driven part, non-systemic motion errors because of interference with external magnetic fields, and limited miniaturization and integration possibilities [2,3,4]. Additionally, the control problem of electromagnetic actuators is challenging because of complex dynamics, internal modeling uncertainties, external disturbances, and high nonlinearity [2]. Electromagnetic actuators may have a compact size, however, they are difficult to scale up, have high energy consumption, and produce heat [3]. The application of several electromagnetic actuators and motors for multi-DOF systems increases structural complexity, and the control of such systems becomes more complicated [5,6]. Therefore, new precise motion devices must be developed to overcome the aforementioned drawbacks.

Piezoelectric actuators and motors are one of the options that meet the requirements of modern mechatronic systems. This type of actuator has a high resolution of motion, short response time, and scalable design, they are magnetic field free, and can achieve MDOF motion using a single actuator [4,7]. Moreover, piezoelectric actuators and motors are gear-free and have a self-locking ability, therefore a simpler structural design of the system can be developed [8]. In comparison with electromagnetic actuators, the most important advantage of piezoelectric actuators is the ability to provide nanometer or sub-micrometer resolution [9]. Control of the piezoelectric actuators is performed by the applied voltage on particular electrodes and evaluation is made by measuring dynamic characteristics of the system, such as displacement, velocity, or acceleration [10]. Flexible design principles allow for the development of piezoelectric motors with single or dual rotors, or for the implementation of motors with several stators [11,12]. However, it must be noted that piezoelectric capacity is changed during the operation of the motor, which causes a severe hysteresis to its input voltage-output displacement relationship, so motion control systems must be implemented [4].

The most common design of MDOF piezoelectric actuators is based on different shapes of beams, such as rectangular [13], cylindrical [14], or conical [15]. Usually, the operating principle of piezoelectric actuators with different beam-type stators is based on bending vibration modes or superposition of several modes including bending and longitudinal modes. Despite the simple design, these actuators can provide up to 3-DOF angular motion of spherical rotor while planar motion is not foreseen. Therefore, the development of piezoelectric robots that can provide both motion types is highly relevant.

Hernando-Gracia et al. reported on a piezoelectric bidirectional planar motion robot [16]. The robot is based on a rectangular beam made from glass and two piezoceramic patches placed at the ends of the beam. Four cylindrical beams are placed at the bottom of the piezoelectric beam and operate as legs of the robot that transfer vibrations of the robot body to the contact surface. The operation principle is based on the superposition of two bending modes that generate traveling wave in the body of the robot. Two harmonic electric signals with shifted phases are used for the excitation. The robot provides bidirectional motion with a speed of up to 100 mm/s while an excitation voltage of 65 V_p-p_ is applied. However, the authors did not foresee the steering option as well as the possibility to rotate the payload that could be placed on top of the robot.

Deng et al. proposed a planar motion robot that is based on four legs driven by two groups of piezo ceramic rings [17]. Each leg bends in vertical and horizontal directions simultaneously when two groups of the piezoceramic rings are excited by applying different excitation schematics and the planar motion of the robot is induced. This operation mode ensures the possibility to obtain micrometer scale positioning for long distances. In addition, the robot can operate in a swinging mode which ensures the possibility to obtain a nano-metric resolution of the motion. Based on numerical and experimental investigations, authors claimed that the robot can achieve a linear velocity of 3.65 mm/s and 3.52 mm/s in X and Y directions, respectively, while an excitation voltage of 400 V_p-p_ and a mechanical load of 19.4 kg was applied. It must be noted that the design of the robot has limited scalability options and excitation of the angular motion of the payload was not foreseen.

Chen et al. reported on piezoelectric angular motion motor with two degrees of freedom [18]. The design of the motor is based on two identical cross-type stators that are formed around the ring and placed in front of each other. A spherical rotor is placed between stators. Eight piezo ceramic patches are placed at the ends of cross-type stators and are used to excite B_11_ and B_12_ vibration modes of elastic rings and to rotate the spherical rotor. The design and operation principle of the motor allows for achieving two 2-DOF motions of the rotor. The motor achieves a rotation velocity of 769.7 deg/s while an excitation voltage of 300 V_p-p_ is applied. The motor has a simple and scalable design and can provide 2-DOF motion. However, the motor cannot provide planar motion of the rotor and is limited to two degrees of freedom.

Yan et al. reported on three degrees of freedom rotary motor [19]. The design of the motor is based on square shaped beam fixed at one end, while at the center of the beam the cylindrical cavity is formed and is used to transfer vibrations of the stator to the spherical rotor. The outside surface of the beam is covered by four piezo-ceramic patches. The operation of the motor is based on the superposition of the second bending and first longitudinal vibration modes of the stator. Vibrations of the motor are excited using three harmonic signals with a phase difference of π/2. The motor provides angular motion of the rotor about three axes while rotation direction is controlled using particular excitation schematics. Experimental and numerical investigation showed that the motor can reach up to 327 RPM rotation speed while the excitation voltage was 200 V_p-p_. The design of the motor is simple and well-scalable. However, it does not foresee the planar motion of the payload and deeper integration of the motor with electronics.

Literature review showed that there are MDOF actuators and motors developed up until now used to drive payload in the plane or obtain angular motion, however, neither of them provide planar and angular motions simultaneously. This paper presents a new design of the 5-DOF robot, the design of which is based on the results of previous investigations [20].

## 2. Design and Operation Principle of the Robot

**Figure 1:**
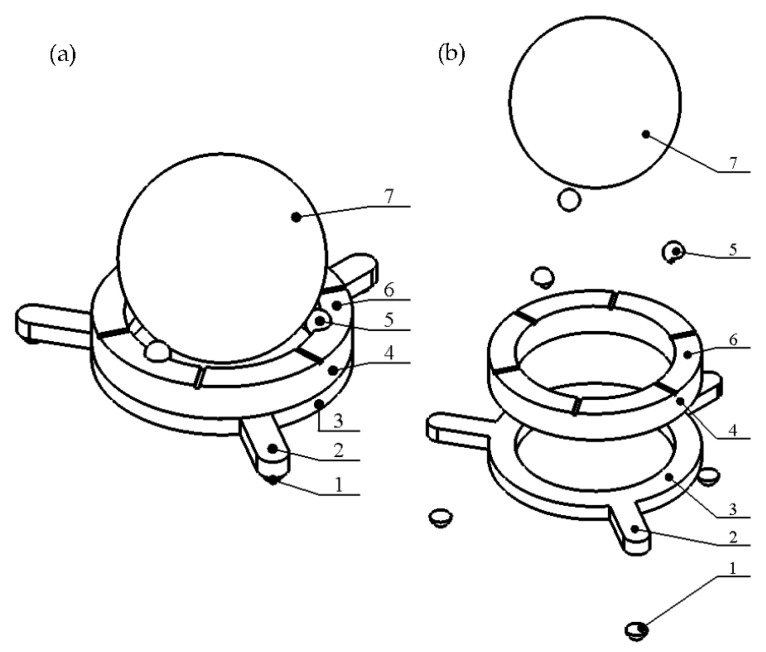
Design of the robot; (**a**)—assembled view; (**b**)—exploded view; 1—alumina oxide contact for planar motion; 2—spike; 3—ring made from stainless steel; 4—piezo ceramic ring; 5—alumina oxide contact for angular motion; 6—segments of the top electrode; 7—spherical payload.

**Figure 2:**
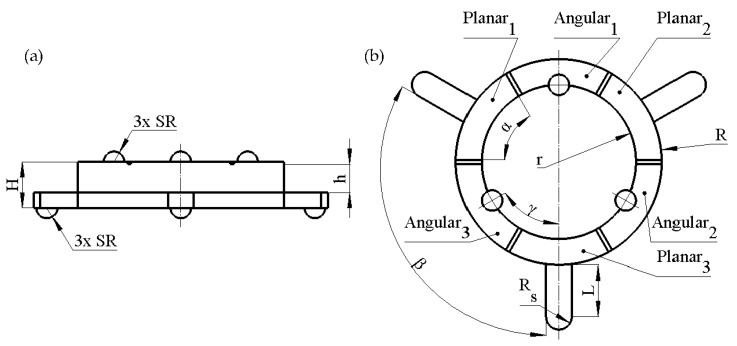
Sketch of the robot; (**a**)—side view; (**b**)—top view; Planar_1_–Planar_3_ are segments of piezo ceramic ring dedicated to control planar motion; Angular_1_–Angular_3_ are segments of piezo ceramic ring dedicated to controlling angular motion.

The proposed piezoelectric robot is composed of a ring-shaped passive layer made from stainless steel and a piezoceramic ring (Figure 1). The top electrode of the piezoceramic ring is divided into six segments and is used to control the direction of planar and angular motion of the payload (Figure 2). The piezoceramic ring is glued on top of the passive layer and is polarized along the thickness of the ring. The ring-shaped passive layer has three spikes oriented in the radial direction of the ring. An alumina oxide ball is glued at the end of each spike of the steel ring and is used as contacting element. The spikes are used to transfer vibrations of the piezoelectric ring to contacting elements and to induce the planar motion of the payload. Additionally, three alumina oxide balls are glued to the top surface of the piezoelectric ring and are used to generate rotational motion of the spherical payload about three axes. Dimensions of the piezoelectric robot are shown in Table 1. The robot has a compact size, simple design, low profile, and good scalability. The total volume of the investigated robot is 3.49 cm^3^ while the total weight of the robot without a payload is 6.15 g.

The operation of the robot is based on the excitation of two vibration modes, i.e., the first bending mode of spikes and the third radial mode of piezoceramic ring. In order to obtain the planar motion of the robot, a single harmonic signal with a frequency equal to the natural frequency of the bending mode of the spike must be applied to the dedicated segment of the electrode (Planar_1_–Planar_3_). As a result, the motion of the robot and payload is generated. The motion direction of the robot is controlled by applying an electric signal to different segments of the electrode.

Angular motion of the payload is generated when electrode segment Angular_1_–Angular_3_ is affected by a harmonic signal with a frequency equal to the natural frequency of the third radial mode of the piezoceramic ring. In this case, alumina oxide contacting elements impacts payload, and the angular motion is generated. In order to obtain the angular motion of the payload about a different axis, a particular segment dedicated to the angular motion must be affected by an electrical signal. In addition, the passive segments of the piezoceramic ring must be short-circuited to avoid possible excitation of parasitic vibrations of the passive contacting elements.

**Figure 3:**
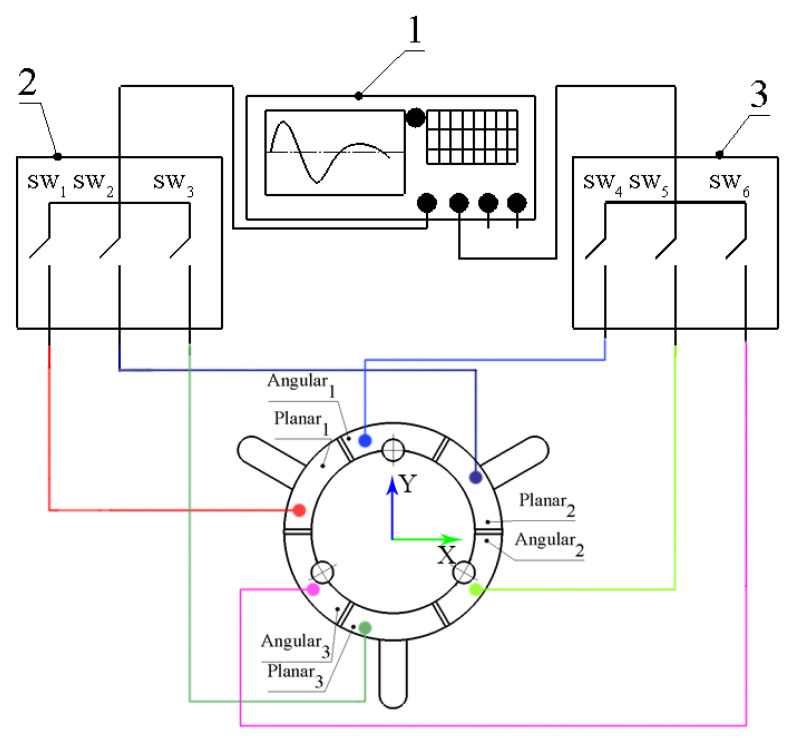
Excitation schematics of the robot; 1—excitation signal source; 2—switching box dedicated to controlling planar motion; 3—switching box dedicated to controlling angular motion.

Special excitation schematics and a switching box are used to control the driving signal (Figure 3). Configuration of the switching box used to control applied signals to the particular electrode is shown in Table 2, where 1 and 0 denote active or passive segments of the top electrode of the piezoceramic ring, respectively.

It can be seen that the motion control of the robot is performed by switching electric signals between electrodes (Table 2). In addition, the control signals used in switch boxes can be combined to obtain angular and planar motions at the same time. Additionally, different algorithms used for planning planar and angular motion trajectories can be applied to obtain synchronous or asynchronous motions of the robot and spherical payload [21]. Moreover, to obtain higher output forces or resolutions, the control algorithm can be set up to use burst type or DC signals.

## 3. Numerical Investigation of the Robot

A numerical investigation was performed to indicate vibration modes suitable for robot operation and investigate the electrical and mechanical characteristics of the robot. The numerical model of the robot was built using Comsol Multiphysics 5.4 (COMSOL, Inc., Stockholm, Sweden) with strict respect to geometrical characteristics shown in Table 1. Material characteristics used to build the model are given in Table 3. Stainless steel DIN 1.4301 was used to build the passive layer of the robot and PIC181 (PI Ceramics, Lederhose, Germany) piezoceramic was used for the piezoceramic ring (Figure 1). Finally, alumina oxide characteristics were used to set up contacting elements placed at the ends of spikes and on the top of piezoceramic ring.

**Figure 4:**
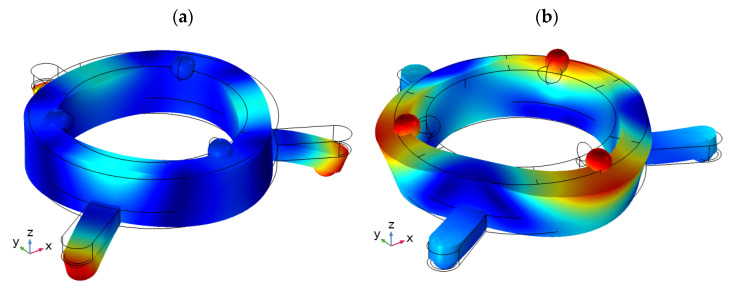
Modal shapes of the robot at the frequency of 28.32 kHz (**a**) and 41.78 kHz (**b**) were used for planar and angular motion, respectively.

Modal analysis of the robot was performed to indicate suitable vibration modes. The payload was simulated as a mass, placed on top of the robot. The electrodes of the piezoceramic ring were set to short circuit condition. No mechanical boundary conditions were applied. Suitable modal shapes were found at the frequency of 28.32 kHz and 41.78 kHz (Figure 4).

When analyzing the modal shapes of the robot, it can be seen that spikes of the ring vibrate at the first bending mode at the frequency of 28.32 kHz. This mode will be used for inducing the planar motion of the robot. The modal shape obtained at the frequency of 41.78 kHz has dominated the third radial vibration mode of the piezoceramic ring and will be used for rotating spherical payload. Moreover, it must be mentioned that the modal shape used for planar motion also induces small vibrations of contact elements used for angular motion located on the top of the piezoceramic ring. Similarly, the contacting elements located on the spikes vibrate when the vibration mode used for angular motion is generated. Therefore, the numerical analysis will be performed to analyze the ratio between vibration amplitudes of the contacting elements used for planar and angular motion.

**Figure 5:**
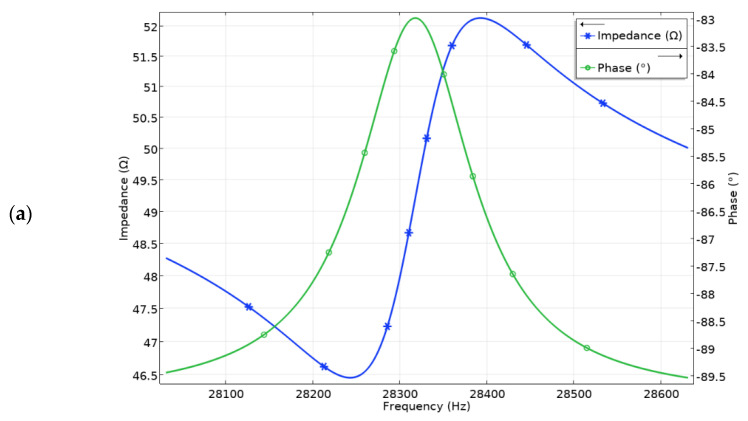

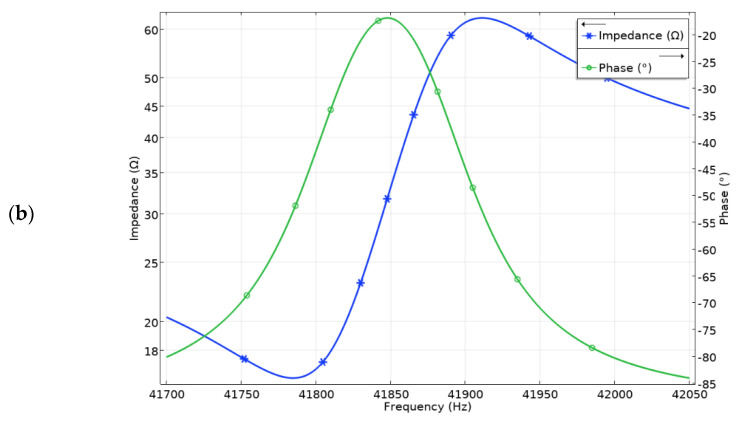
Impedance and phase frequency characteristics of the robot: (**a**)—for planar motion; (**b**)—for angular motion.

The next step of the numerical study was to calculate the impedance and phase frequency characteristics of the robot. The results are shown in Figure 5. It was found that the resonant frequency of vibrations used for planar and angular motion is 28.25 kHz and 41.86 kHz, respectively. The differences between natural and resonant frequencies are 7 Hz and 8 Hz for planar and angular motions, respectively. Minor differences between natural and resonance frequencies occur due to a mismatch in electrical boundary conditions during calculations. However, the mismatches are minor and do not have a notable influence on further calculations. Analyzing the results of impedance-phase characteristics in the frequency domain it can be found that the damping of the robot is notable. It is affected by a payload that was modeled as a distributed mass located on the top surface of the robot.

**Figure 6:**
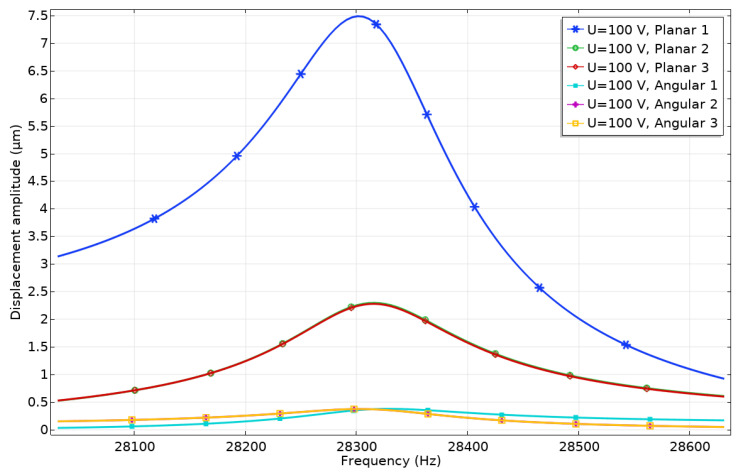
Amplitude—frequency characteristics of the contacting points when electrode Planar_1_ is affected by the voltage of 100 V_p-p_.

Harmonic response analysis was performed to study displacement amplitudes of contacting elements while electrodes used to generate planar or angular motion were excited by harmonic signals. The ratio between vibration amplitudes of the active and passive contacting element was investigated as well. Firstly, electrode Planar_1_ was affected by the harmonic voltage of 100 V_p-p_ while other electrodes (Planar_2_, Planar_3,_ and Angular_1_–Angular_3_) were set to short circuit conditions. The results are given in Figure 6, where the amplitudes of contact points vibrations in the frequency domain are shown.

It can be seen that displacement amplitudes of contact vibrations located at the end of the spike obtained by exciting Planar_1_ electrode reached the value of 7.49 μm or 74.9 nm/V. On the other hand, it must be noted that the vibration amplitude of the passive spikes is 2.29 μm or 22.9 nm/V and 2.26 μm or 22.6 nm/V. These displacements occur due to vibrations transferred from the active electrode of the piezoceramic ring to the passive one and can be named parasitic vibrations. The ratio between displacement amplitudes of active and passive spikes is 3.3, so it will have a minor influence on the direction and accuracy of planar motion. Vibration amplitudes of the contact points located on the piezoceramic ring and used for angular motion have much smaller values and reach values in the range of 0.37–0.38 μm or 3.7–3.8 nm/V. The difference between the vibration amplitudes of the passive contacts related to planar and angular motions is approximately six times while the difference between the vibration amplitude of active planar contact and passive angular contacts is up to 20 times.

**Figure 7:**
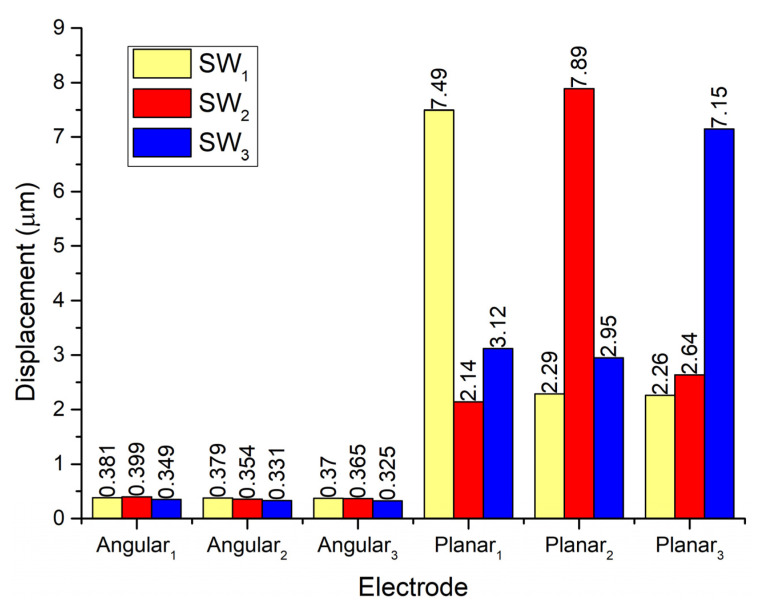
Comparison of displacement amplitudes while electrodes Planar_1_–Planar_3_ are excited.

In order to analyze vibration amplitudes of the contact elements used for planar motions and their influence on remaining contacts, a numerical investigation was performed while Planar_2_ and Planar_3_ electrodes were affected by excitation signals. A summary of the results is given in Figure 7.

It can be seen that displacement amplitudes of contacts located at the ends of spikes have similar vibration amplitudes when the corresponding electrode used for planar motion is excited. The difference between amplitudes is around 10%. In addition, during the excitation of planar motion, vibrations of passive spikes can be observed as well. The difference between vibrations of active and passive spikes is 66.5% while the difference between amplitudes of active spikes and passive contacts used for angular motion is up to 95%. Results show that during the excitation of planar motions the passive spikes can have a minor influence on the motion direction of the robot. On the other hand, vibrations of contacting elements used for angular motion also occurs during the excitation of planar motion. Amplitudes of these vibrations are much smaller compared to vibrations of planar contacts but they can influence the angular position of the payload. The errors of planar and angular motion can be compensated using a motion control system.

**Figure 8:**
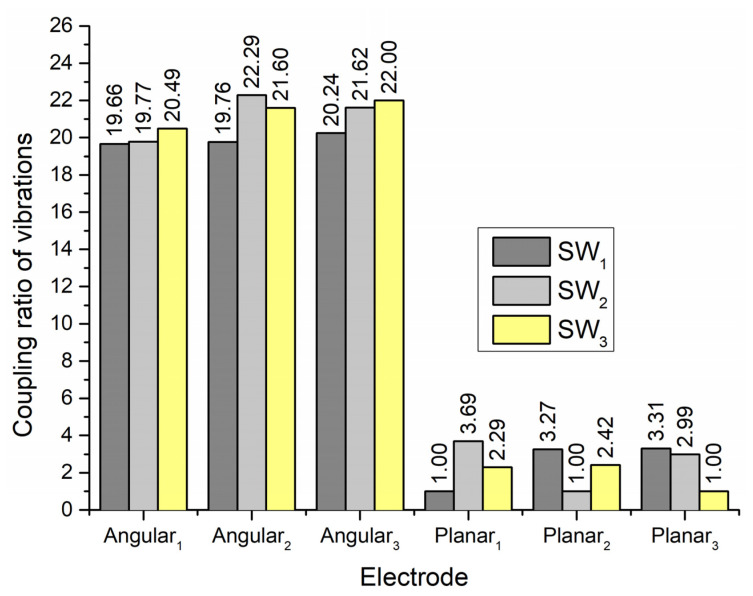
Vibration coupling ratios when electrodes Planar_1_–Planar_3_ are excited.

To fully estimate the vibration characteristics of the contacts and their possible influence on the planar and angular position of the payload, the coupling ratio of vibration of active and passive contacts was calculated. The coupling ratio is calculated as the ratio between displacement amplitudes of contact elements located on the active and passive electrodes. The results are given in Figure 8.

It can be seen that coupling ratios between vibration amplitudes of planar and angular contacts points when one of the planar electrodes is affected by an excitation signal varies from 19 to 21. Therefore, vibrations for planar motion will have a minor influence on the angular position of the payload. On the other hand, coupling ratios between vibration amplitudes of contacts used to generate planar motion vary from 2.3 to 3.3. Therefore, it can be found that vibrations of contacts related to planar motion could influence planar motion accuracy and direction.

**Figure 9:**
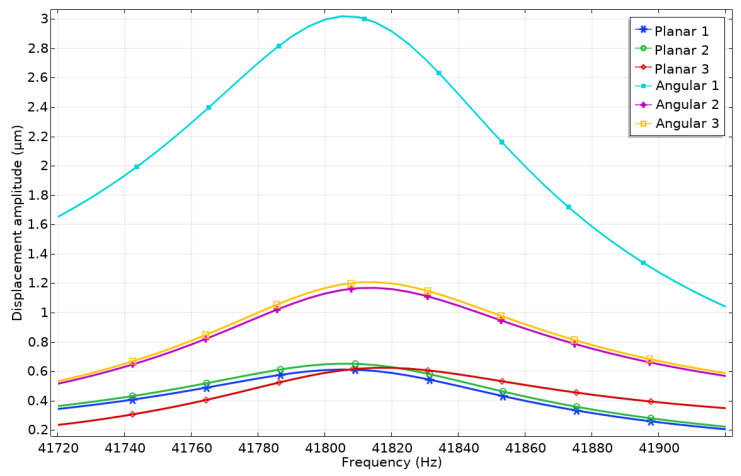
Displacement—frequency characteristics of the robot when the voltage of 100 V_p-p_ is applied to electrode Angular_1_.

A numerical investigation dedicated to analyzing displacement amplitudes of contacts used for the angular motion of the payload was performed. In addition, the vibrations of passive angular and planar contacts will be analyzed. During the first numerical experiment, the electrode Angular_1_ was applied by the harmonic electric signal of 100 V_p-p_ while the remaining electrodes were set to short circuit condition. The results are shown in Figure 9.

It can be seen that the vibration amplitude of contact located on the Angular_1_ electrode reaches a value of 3.01 μm while contacts located on electrodes Angular_2_ and Angular_3_ have amplitudes of 1.16 μm and 1.2 μm, respectively. These displacements can be named parasitic vibrations because they can influence motion direction as well as the accuracy of angular position. The differences between vibration amplitudes of active and passive contacts are up to 61.5%. In addition, it can be noticed that displacement peaks have slight drift, which occurs due to differences in electrical boundary conditions between electrodes, i.e., Angular_1_ is affected by excitation signal while Angular_2_ and Angular_3_ are set to short circuit conditions. Additionally, it can be found that contacts located at the ends of spikes vibrate with displacement amplitudes of 0.62–0.65 μm. The difference between displacement amplitudes of active angular and passive planar contacts is up to 79% so the influence of passive contact vibrations will be minor and can be compensated via control and driving algorithms.

**Figure 10:**
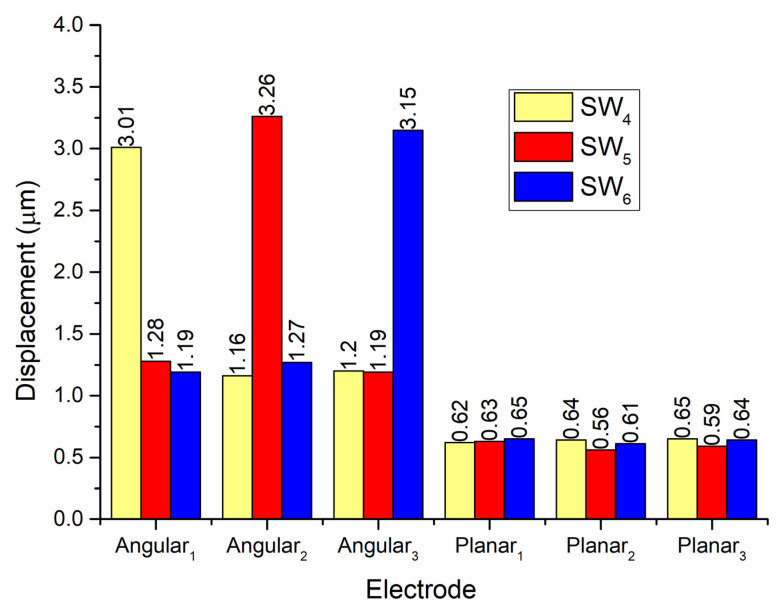
Comparison of displacement amplitudes while electrodes Angular_1_–Angular_3_ excited by excitation signal consistently.

Investigation of displacement amplitudes of contact elements located on Angular_2_ and Angular_3_ electrodes was performed as well. Results are given in Figure 10. It can be seen that differences between displacement amplitudes of contacts used for inducing angular motion do not exceed 7.66%. It shows that the angular motion of the payload in different directions will have similar characteristics. The difference between displacement amplitudes of passive angular contacts does not exceed 9.4%. It means that vibrations of passive contacts will have a minor influence on the angular positioning of the payload. Moreover, displacement amplitudes of contact points located at the ends of the spikes have almost the same amplitudes while the angular motion of the payload is generated.

**Figure 11:**
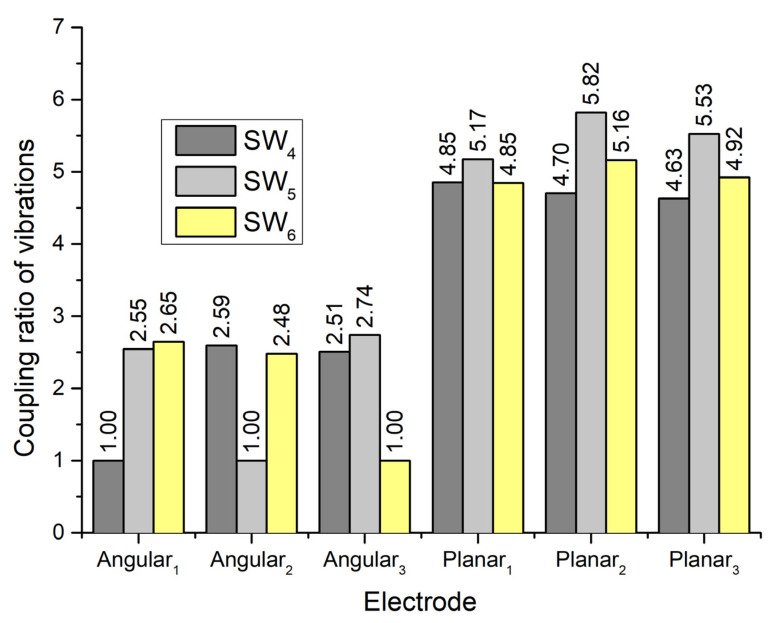
Vibration coupling ratios when electrodes Angular_1_–Angular_3_ are excited.

The coupling ratio between vibration amplitudes of passive and active contacts was calculated as well. The results are given in Figure 11. It shows that the coupling ratio between active and passive angular contacts is around 2.6 while the ratio between contacts of angular and planar motions is around 5. So, the generation of angular motion of the payload has a minor influence on the planar positioning of the robot.

Considering the results of the calculation it can be concluded that displacement amplitudes of contacts are suitable for planar and angular motion generation. Moreover, vibrations amplitudes of passive contacts are significantly smaller and will have a minor influence on the disturbance of planar or angular motion direction.

**Figure 12:**
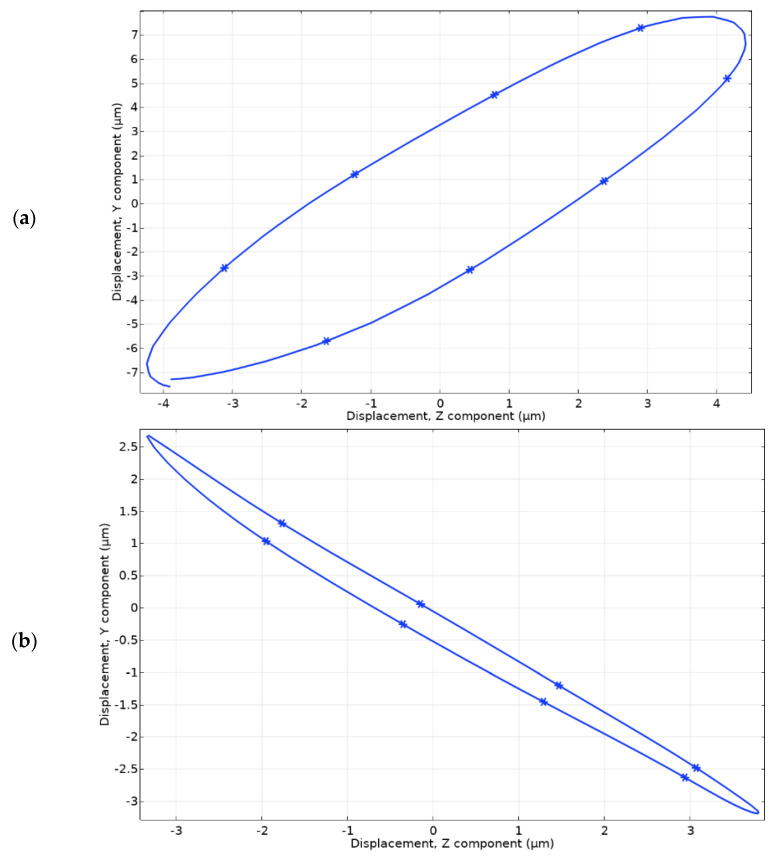
Motion trajectories of the contacting points located at the end of spike and driven by Planar_1_ electrode (**a**) and motion trajectory of contact point located on top of Angular_1_ electrode (**b**).

A numerical study of motion trajectories of planar and angular contact points was performed. For this purpose, a time-dependent study was used. Simulation time was equal to one period of vibrations at the resonant frequency. Boundary conditions were set the same as in previous cases. The excitation voltage of 100 V_p-p_ was applied to the corresponding planar electrode Planar_1_ or angular electrode Angular_1_. The results are shown in Figure 12.

It can be seen that the motion trajectory of contact located at the end of the spike has an elliptical trajectory. The projection of the major axis of trajectory to the X axis is 8.7 μm while the projection of the major axis to the Y axis is 13.25 μm. In addition, the projection of the minor axis of motion trajectory to the X axis is 2.15 μm while the projection to the Y axis is 4.11 μm. Considering the shape of the motion trajectory of contact, it can be stated that the planar motion of the robot with payload will be generated during the excitation of the Planar_1_ electrode.

Analyzing the motion trajectory of the contact point located on top of the Angular_1_ electrode it can be seen that it has an elliptical trajectory as well. The projection of the major axis of motion trajectory to the X axis is 7.14 μm while to the Y axis is 5.86 μm. In addition, the projection of the minor axis of motion trajectory to the X axis is 0.3 μm while the projection to the Y axis is 0.63 μm. Considering the shape of the motion trajectory as well as the length of the projections of the minor and major axis it can be stated that the angular motion of the payload will be generated during the excitation of the Angular_1_ electrode.

## 4. Experimental Investigation of the Robot

**Figure 13:**
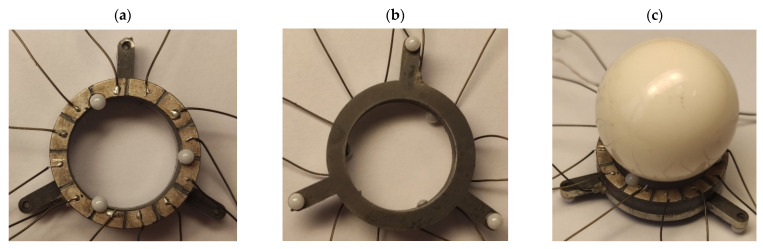
Prototype of the robot; (**a**)—top view; (**b**)—bottom view; (**c**)—side view with spherical payload.

The prototype of the piezoelectric robot was made to perform an experimental investigation and validate the results of numerical modeling (Figure 13). The same materials and dimensions of the robot were used for the prototype as it was used for the numerical model (Table 1 and Table 3). Planar and angular electrodes were divided into two equal sections to have more flexible control and driving opportunities for the robot. However, an experimental investigation was made by applying an excitation scheme as shown in Figure 3. So, two smaller electrodes were paired for this purpose.

Firstly, impedance–frequency characteristics of the robot were measured. SinePhase impedance analyzer 16,777 k (SinePhase, Mödling, Austria) was used for measurement. Electrical boundary conditions were the same as it was during numerical modeling.

**Figure 14:**
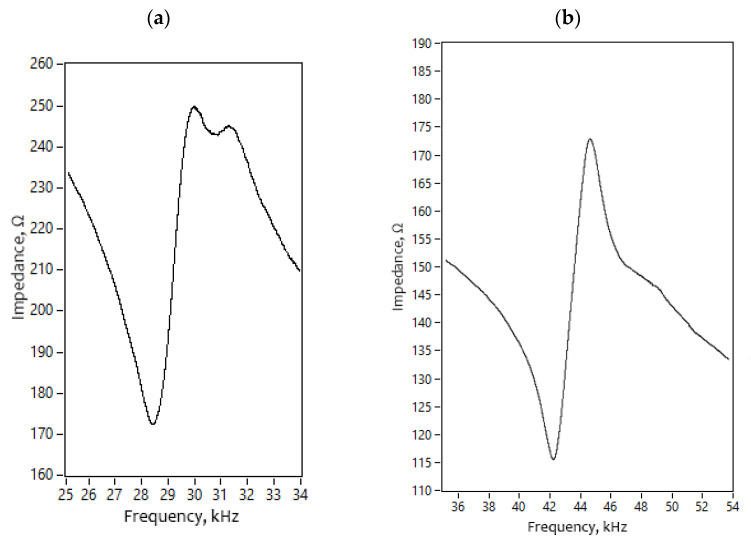
Impedance—frequency characteristics of the robot; (**a**)—for planar motion; (**b**)—for angular motion.

It can be seen that the resonance frequency used for the planar and angular motion of the robot was obtained at 28.45 kHz and 42.17 kHz, respectively (Figure 14). The difference between modeled and measured frequencies is 130 Hz or 0.5% for planar motion and 390 Hz or 1% for angular motion. The mistakes are caused by minor differences in boundary conditions, material characteristics as well as manufacturing errors. However, it can be stated that the results of numerical and experimental investigations are in good agreement and further experimental investigations can be performed.

**Figure 15:**
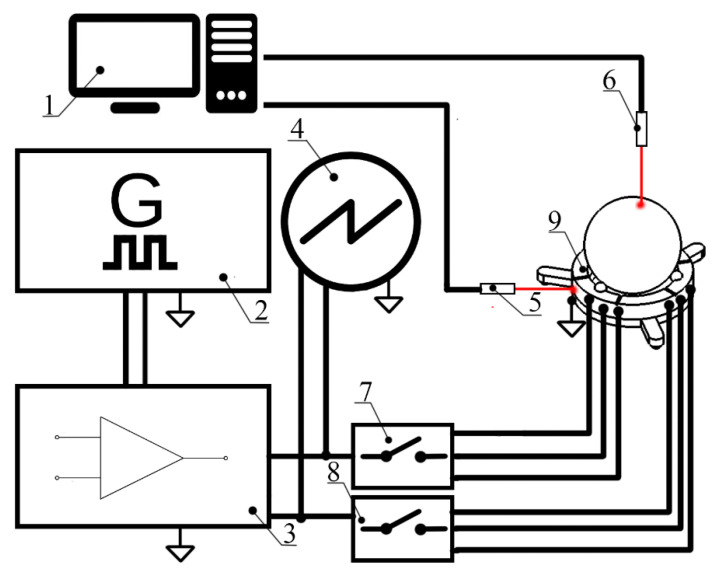
Schematics of the experimental setup; 1—computer; 2—signal generator with two independent channels; 3—power amplifier; 4—oscilloscope; 5—laser displacement sensor for planar motion measurements; 6— tachometer for angular motion measurements; 7—switch box for planar motion control; 8—switch box for angular motion control; 9—the prototype of robot.

Planar and angular velocities were measured as the next step of the experimental investigation. For this purpose, an experimental setup was built (Figure 15).

The experimental setup included a computer, a function generator WW5064 (Tabor Electronics, Nesher, Israel), a power amplifier PD200X4 (Piezo Drive, Shortland, Australia), an oscilloscope DL2000 (Yokogawa, Tokyo, Japan), a displacement sensor ILD 2300 (Micro-Epsilon, Ortenburg, Germany), tachometer DT210 (Nidec-Shimpo, Tokyo, Japan) and self-made switch boxes.

**Figure 16:**
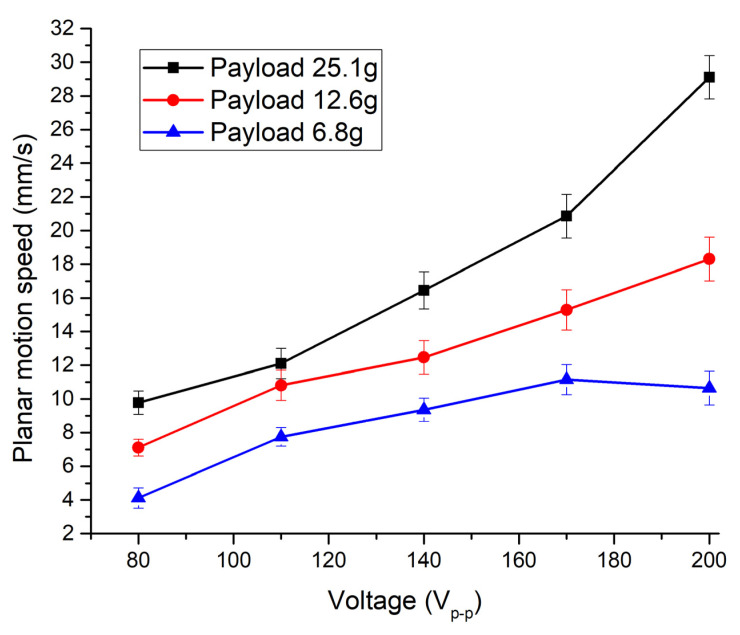
Dependence of planar velocity from excitation voltage when different payloads are applied.

Firstly, the planar velocity of the robot was measured when electrode Planar_1_ was affected by excitation voltages in the range from 80 V_p-p_ to 200 V_p-p_ with the step of 20 V_p-p_. The frequency of the signal was set following impedance–frequency characteristics (Figure 14). In addition, the measurement was performed by applying different masses of spherical payloads, i.e., 6.8 g, 12.6 g, and 25.1 g. The results of the measurements are given in Figure 16. It can be seen that a maximum planar speed of 29.11 mm/s was obtained when the Planar_1_ electrode was excited by the voltage of 200 V_p-p_ and the mass of the payload was 25.1 g. At these conditions, the maximum planar speed per one volt reached 0.145 mm/s/V_p-p_. On the other hand, the lowest planar speed was obtained while the Planar_1_ electrode was affected by an 80 V_p-p_ excitation signal with a payload of 6.8 g, and as a result planar velocity of 4.12 mm/s or 0.05 mm/s/V_p-p_. was obtained. In addition, it can be stated that planar velocity has an almost linear dependence on excitation voltage.

**Figure 17:**
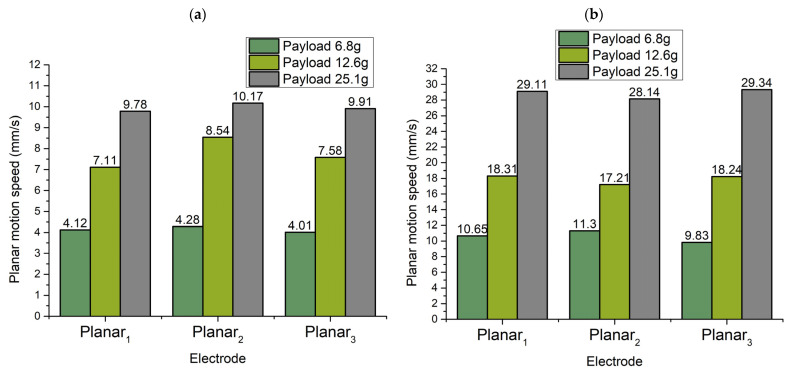
Summary of minimum and maximum planar motion speeds while different payloads are applied to the robot; (**a**)—minimum planar motion speeds while different payloads are used; (**b**)—maximum planar motion speeds while different payloads are used.

To summarize the velocities of the planar motion of the robot, the same measurements were performed with other electrodes as in the case of the Planar_1_ electrode. The summary of maximum and minimum planar velocity is represented in Figure 17 when Planar_2_ and Planar_3_ electrodes were affected by the excitation signal.

Minimum (Figure 17a) and maximum (Figure 17b) values of planar speeds were obtained at a voltage of 80 V_p-p_ and 200 V_p-p_, respectively. The differences between minimum values of the planar speeds when the different payload is used and different electrodes are excited do not exceed 12%. Moreover, it can be seen that minimum planar motion speeds depend on payload values. An increment of friction force between contacting surfaces leads to higher planar velocity. On the other hand, the differences between maximal values of the planar speeds when the different payloads are used and different electrodes are excited do not exceed 14%. In general, it can be stated that planar motion velocity in different directions and with different payloads depends on payload mass.

**Figure 18:**
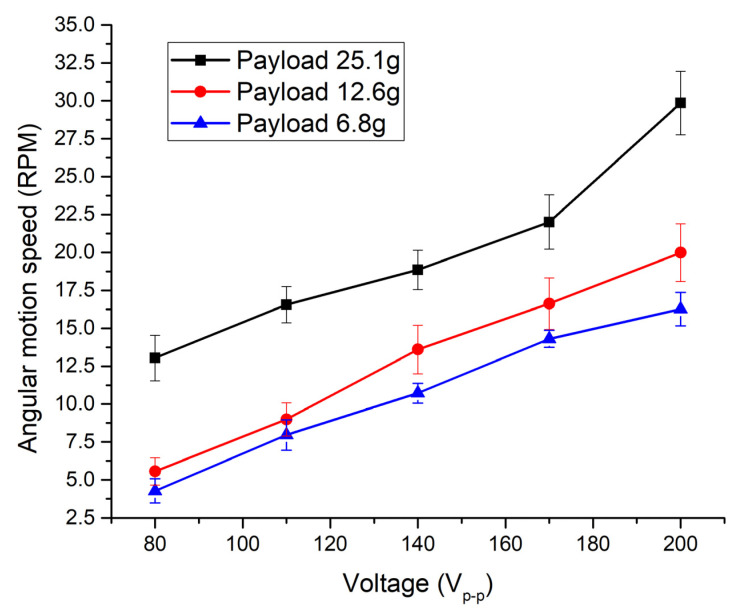
Dependence of angular velocity from excitation voltage when different payloads are applied.

The next step of the experiment was dedicated to the measurement of angular velocity. The same experimental setup was used as shown in Figure 15. Firstly, angular velocity of the spherical payload was measured when electrode Angular_1_ was affected by excitation voltages in the range from 80 V_p-p_ to 200 V_p-p_ with the step of 20 V_p-p_. Three spherical payloads were used for measurement, i.e., 6.8 g, 12.6 g, and 25.1 g. Results are given in Figure 18.

The lowest angular speed was obtained when a payload of 6.8 g was applied and the excitation voltage was set to 80 V_p-p_. The minimum angular speed was 4.27 RPM or 53.3 × 10^−3^ RPM/V_p-p_. On the other hand, the highest angular speed of 29.58 RPM or 147.9 × 10^−3^ RPM/V_p-p_ was obtained when the Angular_1_ electrode was affected by the voltage of 200 V_p-p_ and the payload was 25.1 g. Additionally, it can be stated that angular velocity has an almost linear dependence on excitation voltage.

**Figure 19:**
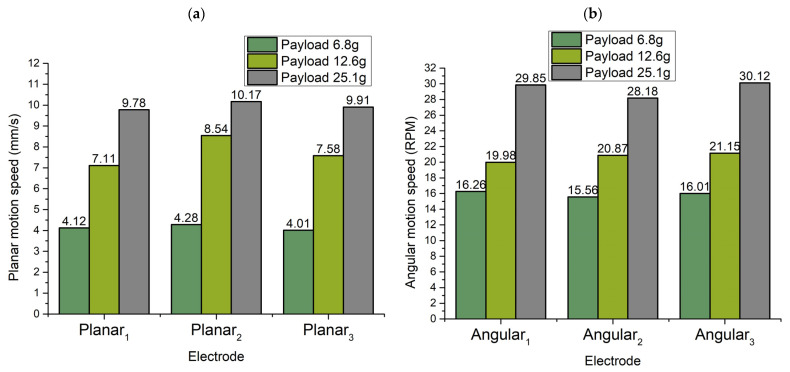
Summary of minimum and maximum angular speed while different payloads are applied to the robot: (**a**)—summary of minimal angular speed; (**b**)—summary of maximum angular speeds.

The same experiments were performed by exciting other electrodes, as was in the case with the Angular_1_ electrode to summarize angular velocities in different directions. Results are shown in Figure 19 when Angular_2_ and Angular_3_ electrodes were affected by excitation signals.

Minimum and maximum values of angular motion were obtained at a voltage of 80 V_p-p_ and 200 V_p-p_, respectively. Additionally, it can be noticed that minimal values of angular velocity have differences that do not exceed 10% while maximum angular velocity has differences that do not exceed 7%. So, it can be stated that angular velocity will have almost the same dynamic characteristics in different directions. In addition, small differences in planar and angular characteristics ensure the possibility to use simple control and driving algorithms.

## 5. Conclusions

A new piezoelectric 5-DOF robot was developed and investigated that can provide planar and angular motion. The robot has a simple and well-scalable design. Results of the numerical investigation showed that the first bending mode of the spikes can be used to obtain the planar motion of the robot and spherical payload. In addition, it was found that the third radial mode of the piezoceramic ring is suitable to induce angular motion of the payload in three different directions. Moreover, numerical investigations revealed that the coupling ratio between vibration amplitudes of passive and active planar contacting elements is up to 3.2 while the coupling between angular active and passive contacts is up to 22. Additionally, similar coupling rations were indicated while the angular motion of the payload are generated. However, to obtain high-accuracy motions, special control and driving algorithms must be developed.

The experimental results showed that differences between calculated and measured resonant frequencies are up to 7%. The robot can provide planar motion speed up to 29.34 mm/s while a 25.1 g payload is used and the voltage of 200 V_p-p_ is applied. A maximum angular velocity of 30.12 RPM was achieved when a payload of 25.1 g was used and a voltage of 200 V_p-p_ was applied. In addition, it was shown that differences in planar and angular velocities in different directions obtained using different contact elements do not exceed 14% when different payloads and voltages are used.

## Figures and Tables

**Table 1:** Geometrical parameters of the robot.

Parameter	Value	Description
R	10 mm	The outer radius of the robot
r	7.5 mm	The inner radius of the robot
SR	1 mm	The spherical radius of alumina oxide contacts
H	4.5 mm	Total height of the robot
h	2.75 mm	Height of piezo ceramic ring
L	5 mm	Length of spikes
R_s_	1.25 mm	The radius of spikes ends
α	60°	Angular value of electrode segment
β	120°	The angular value between spikes
γ	60°	Angular value of top contacts

**Table 2:** Control signals of the actuator.

Motion Direction	Motion Type					
	Planar			Angular		
	SW_1_	SW_2_	SW_3_	SW_4_	SW_5_	SW_6_
0°	1	0	1	1	0	0
120°	1	1	0	1	1	0
240°	0	0	1	0	0	1

**Table 3:** Materials properties used in the model.

Material Properties	Stainless Steel DIN 1.4301	PI Ceramics PIC181	Aluminum Oxide Ceramic
Density, [kg/m^3^]	8000	7800	3980
Young’s modulus, [N/m^2^]	193 × 10^9^	7.6 × 10^10^	41.9 × 10^10^
Poisson’s coefficient	0.29	0.34	0.33
Isotropic structural loss factor	0.02	-	0.2 × 10^−3^
Relative permittivity	-	ε_11_^T^/ε_0_ = 1200 ε_33_^T^/ε_0_ = 1500	-
Elastic compliance coefficient [10^−12^ m^2^/N]	-	S_11_^E^ = 11.80 S_33_^E^ = 14.20	-
Elastic stiffness coefficient c_33_^D^, [N/m^2^]	-	16.6 × 10^10^	-
Piezoelectric constant d_33_ [10^−12^ m/V]	-	265	-
Piezoelectric constant d_31_ [10^−12^ m/V]	-	−120	-
Piezoelectric constant d_15_ [10^−12^ m/V]	-	475	-
